# Aerobic exercise and yoga improve neurocognitive function in women with early psychosis

**DOI:** 10.1038/npjschz.2015.47

**Published:** 2015-12-02

**Authors:** Jingxia Lin, Sherry KW Chan, Edwin HM Lee, Wing Chung Chang, Michael Tse, Wayne Weizhong Su, Pak Sham, Christy LM Hui, Glen Joe, Cecilia LW Chan, P L Khong, Kwok Fai So, William G Honer, Eric YH Chen

**Affiliations:** 1 Department of Psychiatry, The University of Hong Kong, Hong Kong, China; 2 Institute of Human Performance, The University of Hong Kong, Hong Kong, China; 3 Department of Psychiatry, University of British Columbia, Vancouver, British Columbia, Canada; 4 Department of Social Work & Social Administration, The University of Hong Kong, Hong Kong, China; 5 Department of Diagnostic Radiology, The University of Hong Kong, Hong Kong, China; 6 Department of Anatomy, The University of Hong Kong, Hong Kong, China

## Abstract

Impairments of attention and memory are evident in early psychosis, and are associated with functional disability. In a group of stable, medicated women patients, we aimed to determine whether participating in aerobic exercise or yoga improved cognitive impairments and clinical symptoms. A total of 140 female patients were recruited, and 124 received the allocated intervention in a randomized controlled study of 12 weeks of yoga or aerobic exercise compared with a waitlist group. The primary outcomes were cognitive functions including memory and attention. Secondary outcome measures were the severity of psychotic and depressive symptoms, and hippocampal volume. Data from 124 patients were included in the final analysis based on the intention-to-treat principle. Both yoga and aerobic exercise groups demonstrated significant improvements in working memory (*P*<0.01) with moderate to large effect sizes compared with the waitlist control group. The yoga group showed additional benefits in verbal acquisition (*P*<0.01) and attention (*P*=0.01). Both types of exercise improved overall and depressive symptoms (all *P*⩽0.01) after 12 weeks. Small increases in hippocampal volume were observed in the aerobic exercise group compared with waitlist (*P*=0.01). Both types of exercise improved working memory in early psychosis patients, with yoga having a larger effect on verbal acquisition and attention than aerobic exercise. The application of yoga and aerobic exercise as adjunctive treatments for early psychosis merits serious consideration. This study was supported by the Small Research Funding of the University of Hong Kong (201007176229), and RGC funding (C00240/762412) by the Authority of Research, Hong Kong.

## Introduction

Cognitive impairments are recognized as a core feature of schizophrenia. These impairments are detectable in the early stages of illness, and are relatively stable in comparison with fluctuating clinical states.^1^ Both cross-sectional and longitudinal follow-up studies demonstrated that cognitive impairments are significantly correlated with functional outcomes with medium effect sizes.^2^

Cognitive impairments are present in both treated and untreated patients, and symptomatic stabilization is usually not accompanied by cognitive improvement.^3^ The largest and most rigorous clinical trial failed to find any advantages of atypical antipsychotics for improvement of cognitive functions.^4^ Besides medications, non-pharmacological treatments for cognitive deficits, including social skills training, cognitive remediation and exercise, are widely explored in schizophrenia. To date, physical exercise has attracted attention for improving cognitive functioning and avoiding medication side effects in psychiatric patients. Apparently robust improvements in clinical symptoms have been reported as a result of engaging in physical exercise. Systematic reviews demonstrated that aerobic exercise can reduce primary symptoms in patients with schizophrenia, and attenuate secondary symptoms such as depression, self-depreciation and social withdrawal.^5^ However, the benefit of physical exercise for neurocognitive impairment in schizophrenia has not received comparable attention.^6^ Recently, a randomized controlled trial investigated the cognitive impacts of aerobic exercise in chronic schizophrenic in patients participating in a 12-week cycling program. A significant improvement in short-term memory was observed, which was associated with an increase in hippocampal volume.^7^

Aside from aerobic exercise, other popular forms of exercise may also have beneficial neurocognitive effects. Yoga is a body–mind exercise with a distinctive meditative component, and is particularly popular with women in Asian cultures. The effects of yoga on symptoms and emotional deficits in schizophrenia have received increased research attention.^8^ Significant reductions in the Positive and Negative Syndrome Scale (PANSS) total score, positive, negative, and general psychopathology subscores were observed in patients with schizophrenia spectrum disorders treated with yoga compared with controls.^9^

The present study aimed to examine a primary hypothesis that both yoga and aerobic exercise would benefit memory in female patients with early psychosis, with a superior effect on attention through yoga. In Hong Kong, yoga is predominantly practiced by women, and we anticipated recruitment would be most successful if the study was limited to women. We also wanted to test whether the findings of Pajonk *et al.* (limited to men with chronic schizophrenia) could be extended to women with early psychosis. As secondary objectives, we hypothesized that exercise-induced improvements in cognition might be correlated with clinical improvement, and that aerobic exercise would be associated with an increase in hippocampal volume.

## Results

**Figure 1:**
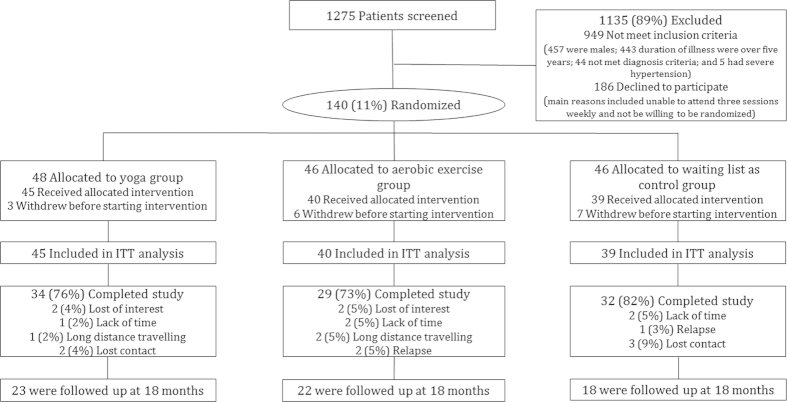
Flow chart of patients through study. IIT, intention to treat.

The numbers and flow of patients through the study appears in Figure 1. One hundred and twenty four patients were included in the final analysis, and 95 completed the 12-week course. The overall dropout rate was 23.4%, and there were no significant differences in the yoga group (24%), the aerobic exercise group (27%), and the waitlist control group (18%).

There were no statistically significant differences in age, years of education, length of illness, marital or occupational status, smoking, substance abuse, or antipsychotic dose between those participants who completed the study and those who dropped out. Non-completers had lower scores in verbal acquisition (19.6 vs. 23.6; *P*=0.02), and retention (12.0 vs. 15.7; *P*=0.01); and had higher baseline PANSS total scores (55.4 vs. 44.8; *P*<0.01), largely related to higher scores on the negative subscale (13.4 vs. 10.4; *P*=0.01) and the general subscale (30.8 vs. 24.8; *P*=0.01). Attendance rates were not significantly different in the yoga group (47%) and aerobic exercise group (58%). Both interventions were observed by an investigator during each session and were carried out at the individual-specific level of activity as described in the Materials and Methods. No adverse events were reported during the study.

**Figure 2:**
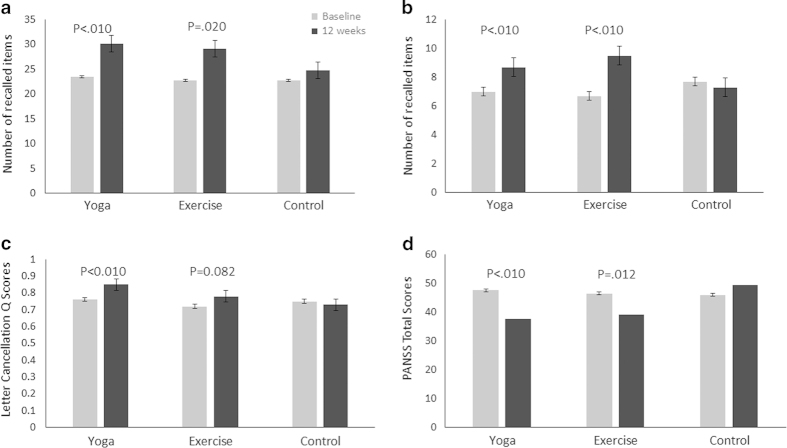
Mean cognitive scores and symptom values for patients in the three groups, according to yhe HKLLT, Digit Span Backwards Test, Letter Cancellation Test and PANSS total scores at baseline and 12 weeks for verbal acquisition (**a**), working memory (**b**), attention (**c**), and symptom severity (**d**).

Primary and secondary outcomes appear in Figure 2, Table 1 and Table 2. When comparing all the three groups with a mixed-model, Group×Time interactions were statistically significant in verbal acquisition and retention, working memory, and attention, measured by HKLLT, Digit Span forwards and backwards tests, and Letter Cancellation test, respectively. When *a priori* comparisons were made between the active intervention groups and the waitlist group, statistically significant improvements were observed in verbal acquisition, working memory and attention for the yoga group; and in verbal retention and working memory for the aerobic exercise group. However, the effects of verbal acquisition (F=10.81, *P*<0.01), working memory (F=9.56, *P*<0.01 in Digit Span Forwards test; F=10.75, *P*<0.01 in Backwards test), and attention (F=7.45, *P*<0.01) for the yoga group, and the effect of working memory (F=6.43, *P*=0.014 in Digit Span Forwards test; F=30.82, *P*<0.01 in Backwards test) for the aerobic exercise remained significant when age, education years, length of illness, and antipsychotic dose were included as covariates. The effect sizes were moderate-to-large for both yoga and aerobic exercise.

The effects on cognition observed at 12 weeks in both intervention groups were maintained at 18 months follow- up. There were no significant changes in verbal acquisition (F=1.42, *P*=0.24), working memory (F=0.07, *P*=0.80), and attention (F=0.65, *P*=0.42) in the yoga group; nor in working memory (F=0.01, *P*=0.92) in the aerobic exercise group at 18 months compared with 12 weeks.

Overall symptom severity improved both in yoga (F=11.87, *P*<0.01) and aerobic exercise (F=6.76, *P*=0.012) groups, largely related to improvement in the general subscale (with age, length of illness and antipsychotic dose as covariates). Negative symptoms improved in the yoga group (F=11.70, *P*<0.01). Depressive symptoms improved in both yoga (F=10.38, *P*<0.01) and aerobic exercise (F=8.79, *P*<0.01) groups after the 12-week intervention. These effects on clinical symptoms were stable at the 18-month follow-up in both intervention groups. Following the 12-week waitlist period, this group participated in yoga or aerobic exercise. The improvements at 18 months were similar as those observed in the 12-week intervention groups.

Both yoga and aerobic exercise significantly improved the health-related quality of life assessed by the SF-36. Both types of exercise significantly improved ‘physical health (F=6.21, *P*=0.015 for yoga; F=15.52, *P*<0.01 for aerobic exercise), and ‘psychological health’ (F=9.37, *P*<0.01 for yoga; F=6.36, *P*=0.015 for aerobic exercise).

We did not find significant changes in either congruent (F=1.3, *P*=0.28) or incongruent (F=1.7, *P*=0.18) conditions of the Stroop Color and Word Tests among three groups. There were no significant changes in Figure Rating Scale (F=0.04, *P*=0.96) for body perception, and Compliance Rating Scale (F=0.53, *P*=0.59) for medication adherence.

When comparing the changes of hippocampal gray matter volumes among three groups using the FreeSurfer Linear Mixed Model, corrected by age and intracranial volume, aerobic exercise was associated with increased hippocampal gray matter volume (F=7.52, *P*=0.01), mainly related to increases in the left hippocampus (F=5.13, *P*=0.03). Yoga did not show significant changes in total hippocampal volume (F=1.07, *P*=0.31).

The change in VO_2_ max/kg over time in all three groups was compared. The Group×Time interaction was not statistically significant between the three groups (F=0.77, *P*=0.47), with a trend of increased VO_2_ max/kg in aerobic exercise groups (7.7%). To examine possible associations between cognitive and fitness changes more closely, we investigated correlations within all subjects in the study. The correlation between the change in cognition and fitness parameters failed to reach statistical significance (total: *r*=0.07, *P*=0.42 for verbal memory, and *r*=0.06, *P*=0.51 for working memory). However, there was a positive correlation between the number of sessions participants attended and the change in working memory (total: *r*=0.26; *P*=0.02).

## Discussion

This clinical trial compared the effects of two different types of exercise on cognition in early psychosis. The most significant findings were the improvements in working memory, occurring in both intervention groups. Our findings are consistent with the results of Oertel-Knochel *et al.,* ^10^ who showed improved performance in working memory and visual learning after a cognitive training program combined with physical exercise. Improvement in verbal retention did not reach statistical significance for either type of exercise intervention when covariates were included. One possible explanation is that retention may be relatively intact in psychotic patients, as reported by Chan^11^ and Paulsen *et al.* ^12^ As mentioned, a study examining the effects of aerobic exercise on cognitive performance demonstrated that a 12-week cycling program could improve short-term memory in men with chronic schizophrenia, accompanied by a significant increase in hippocampal volume.^7^ However, differences in gender and the stage of illness between our study and Pajonk *et al.* may contribute to the inconsistency.

Currently available evidence for the effects of yoga on cognition in psychosis patients is limited. Previous studies mainly focused on the impacts of yoga on symptoms and social functioning. We observed improved verbal acquisition and Letter Cancellation test scores after 12-week of yoga training, but not aerobic exercise, indicating that yoga may yield superior effects on verbal learning and attention. These results were consistent with a previous study of patients with major depression, which found yoga practitioners significantly improved attention span compared to participants treated only with antidepressant medication.^13^ Findings from imaging studies seem to lend support to these hypotheses.^14^

The changes of VO_2_ max/kg in both intervention groups failed to be statistically significant in the present study. However, there was a trend towards increased fitness in the aerobic exercise group. It is not surprising that yoga did not improve VO_2_ max because it demands less cardiovascular fitness as a mind-body exercise. Fitness testing depends on subjective effort as well as actual fitness. Variable effort could result in apparent reductions in VO_2_ max/kg if patients had less motivation at the fitness testing session. These challenges were also mentioned in a systematic review and meta-analysis.^15^ Changes in measures of fitness could also be complicated by antipsychotic medication treatment. Antipsychotic drugs with cardiac and peripheral vascular effects blunt the acute effects of exercise on cardiac stroke volume and on cardiac output.^16^ The small sample size (20 in the yoga group, 17 in the aerobic exercise group, and 11 in the control group) with completed fitness data should also limit the interpretation of the fitness results.

Colcombe *et al.* suggested that regular exercise resulted in greater brain plasticity, adaptability and efficiency by increasing cortical blood flow, modulating synaptic connections, and developing new neurons.^17^ A vast amount of evidence in both animals^18^ and humans^5^ demonstrated effects of physical exercise on the hippocampus, which has an important role in memory. Our study found a small increase in hippocampal gray matter volume in the aerobic exercise group, consistent in direction although more modest in size compared with that reported in chronic male patients. The mechanisms of improvements in attention by yoga may be related to particular characteristics of yoga practice. Yoga emphasizes mental concentration and the control of body movements, which could contribute to alterations in brain structures and functions leading to the cognitive enhancements.^19^ Slow-motion body movement is thought to be able to capture the mind's attention, and body postures in yoga sharpen the sensory awareness.

Both types of exercise also demonstrated great improvements on general symptoms. These findings are consistent with previous studies of schizophrenia patients.^9^ The statistically non-significant effects on positive symptoms in the current study could be due to the relatively mild and stable psychotic symptoms at baseline. Furthermore, both yoga and aerobic exercise had a statistically significant benefit on depression, which was reported in several previous clinical studies.^20^ Possible biological mechanisms for exercise-induced antidepressive effects include increased beta endorphins, and changes of endogenous opioid peptide neurotransmitters.^20^ Psychological mechanisms, including improved sleep, increased sense of self-efficacy, increased feelings of mastery and reduced negative thoughts, were suggested to be responsible for the effects on depression.^21^

Dropout analyses showed that non-compliant patients had larger deficits in cognition and were more severely ill. It demonstrated that individuals with worse cognitive function or greater symptoms were less likely to benefit from exercise, which would be relevant to note as a limitation that the tolerability to adhere to this kind of intervention may not be comparable across all individuals. There were no significant differences in adherence rates between yoga (58%) and aerobic exercise (47%). The attendance rate was not ideal in our study, but comparable or superior to other community-based studies. The challenges created by dropouts and sometimes low attendance rates support the suggestion by Vancampfort *et al.* that motivational techniques need consideration to improve exercise adherence in patients with psychosis.^22^ The study was limited to women, none had secondary substance abuse and very few were smokers. Further study in a more heterogenous cohort is needed to clarify the extent to which the findings may be generalizable. Finally, although every effort was made to avoid unblinding, it is always possible that blinding may have been compromised in some cases.

The potentially confounding variables including antipsychotic dose were controlled for statistically in the analyses, and no complicating effects were apparent. Arguably, patients in the waitlist group may not serve as an adequate control because of less social contact compared with the patients of the intervention groups. Regular psychoeducation sessions for the waitlist group in future studies could help control for this possibility. Follow-up study is needed to examine whether these benefits by exercise can be maintained in a long-term period. With the benefits in cognition and symptoms, and the advantages of being low-cost and have few if any side-effects, both aerobic exercise and yoga could have potential clinical significance and value as adjunctive therapies for patients with psychosis.

## Materials and methods

### Participants and randomization

This was a single blind, randomized, control study conducted in Hong Kong from Oct 2010 to May 2014. All participants were recruited from the Early Assessment Service for Young People with Psychosis (EASY) program in Hong Kong. Only female patients diagnosed with schizophrenia, schizoaffective disorder, schizophreniform disorder, brief psychotic disorders, psychosis not otherwise specified and delusional disorder (according to the DSM-IV) within 5 years of onset were approached to participate. Those with severe physical illness or other contraindications to exercise according to the American College of Sports Medicine guidelines were excluded. Participants ranged from 16 to 60 years of age (mean (s.d.): 24.6 (7.6) years), with no difference in mean age between groups (Table 3). Sample size was estimated from previous studies, where memory improvement was demonstrated after aerobic exercise intervention, incorporating a two-sided 5% significance level and a power of 80% together with an anticipated dropout rate of 30%. A randomization list was created using a random number generator. The random list had a block size of 12 (i.e., for every 12 participants, 4 would be assigned to the yoga group, 4 to the aerobic exercise group and 4 to the control group). This sequence of randomization was continued for all 140 subjects. The randomization list was concealed from research staff involved in assessment. All patients attended an outpatient clinic and were provided with protocol-based case management intervention. There were no statistically significant differences in years of education, length of illness, PANSS and CDS total scores at the baseline amongst the three groups. Antipsychotic medications were administered as monotherapy (*n*=109) or polypharmacy (*n*=8). The frequently used antipsychotics were risperidone (26.5%), olanzapine (20.5%), quetiapine (17.1%), aripiprazole (13.7%), amisulpride (7.7%), and clozapine (5.1%). Five patients did not take any antipsychotic medication during the study. The protocol was approved by the Institutional Review Board of the University of Hong Kong. All participants provided written informed consent.

### Study procedure

Patients were randomized into three groups: (i) integrated yoga therapy group (*n*=48), (ii) aerobic exercise group (*n*=46), and (iii) waitlist control group (*n*=46; Figure 1). Two certified coaches (with MSc-Exercise Science or PhD training) conducted aerobic exercise and yoga training, and both were blinded to the assessment results. Two research assistants carried out the assessments and were blinded to treatment allocation and the block size of randomization. The research assistants who performed assessments were forbidden to discuss information related to group allocation, participants’ schedule, or activities during the assessment interviews. The assessment interviews were tape-recorded for confirmation.

### Measures

The outcome measures included fitness measures, cognitive performances, clinical assessment for symptoms, structural imaging, measures of body perception, medication adherence and quality of life (Table 3). All the measures have been used in clinical population with good validity and reliability, and were conducted before and after the study period (Supplementary Information). All participants were followed up for 18 months and were assessed again for cognition and clinical symptoms.

### Intervention programs

The intervention programs included integrated yoga therapy and aerobic exercise (walking and cycling). Both programs lasted 60 min for each session, and were held three times a week for 12 weeks (Supplementary Information).

### Statistical analysis

Data analysis was based on the Intention-to-Treat (ITT) method that included all available data in a mixed-model analysis with a repeated-measures approach including an unstructured variance matrix (Table 4). This strategy was based on the assumption that data were missing at random.^23^ Differences between the three intervention groups over time (baseline and 12 weeks) were assessed with a Group×Time interaction term. Four primary outcome measures (verbal acquisition, verbal retention, Digit Span backwards test, Letter Cancellation test Q score) were first analyzed by including all three groups (aerobic exercise, yoga, and waitlist) in analyses, and setting the *P* value at 0.016. For analyses meeting this criterion of statistical significance, follow-up, *a priori* comparisons of the active intervention groups with the waitlist group were carried out with the same strategy. The *P* value for statistical significance in these analyses was also set at 0.016. Secondary outcome measures of symptom severity were carried out comparing each of the two active intervention groups with the waitlist group, and correction for multiple testing was carried out with *P* value at 0.016. Age, years of education, length of illness, and antipsychotic dose (CPZ equivalents) were included as covariates for cognitive data analysis; age, length of illness, and antipsychotic dose were included as covariates for clinical data analysis. Bivariate correlations were used to analyze whether the changes in cognition were associated with the number of sessions attended, or with the amount of change of aerobic fitness.

For statistical analysis of imaging data, following segmentation that included use of a multi-atlas technique (Supplementary Information) hippocampal volume data were analyzed with linear mixed effects Models^16^ in the Freesurfer package. This approach to modeling includes all participants with a baseline scan (whether or not an outcome scan was obtained), and takes into account time between baseline and outcome scan, age and intracranial volume.

## Figures and Tables

**Table 1:** Measures of cognition, clinical symptoms, quality of life, and hippocampal volume at baseline, after 12 weeks intervention and at 18-month follow-up

	*Yoga group*			*Aerobic exercise group*			*Waitlist control group*		
	*Baseline*	*12 weeks*	*18 months*	*Baseline*	*12 weeks*	*18 months*	*Baseline*	*12 weeks*	*18 months*
*Cognition*									
Sample size	45	38	23	40	31	22	39	33	18
HKLLT-acquisition, mean (s.d.)	23.4 (6.1)	30.1 (6.6)	28.1 (6.3)	22.7 (8.0)	29.1 (8.2)	28.9 (8.2)	22.7 (7.6)	24.7 (8.1)	29.6 (6.7)
HKLLT-retention, mean (s.d.)	15.2 (6.1)	19.8 (6.0)	18.4 (6.5)	15.6 (5.4)	21.3 (7.1)	19.6 (7.5)	14.5 (7.0)	16.5 (7.2)	19.1 (6.9)
Digit span forwards test, mean (s.d.)	11.7 (2.3)	12.7 (1.8)	12.4 (2.0)	12.1 (1.8)	13.1 (1.2)	12.8 (1.1)	12.3 (1.8)	12.3 (2.1)	13.4 (0.9)
Digit span backwards test, mean (s.d.)	7.0 (3.0)	8.7 (3.0)	8.5 (3.4)	6.7 (2.5)	9.5 (3.0)	9.4 (3.3)	7.7 (3.3)	7.3 (2.7)	9.2 (3.0)
Letter cancellation test, Q score, mean (s.d.)	0.76 (0.14)	0.85 (0.18)	0.82 (0.15)	0.72 (0.21)	0.78 (0.19)	0.79 (0.21)	0.75 (0.15)	0.73 (0.17)	0.84 (0.13)
Stroop test congruent condition, correct items mean (s.d.)	110.3 (2.6)	111.1 (1.8)	110.9 (1.1)	110.0 (2.7)	110.9 (2.5)	110.6 (1.7)	110.5 (1.6)	110.4 (1.5)	111.4 (1.0)
Stroop test incongruent condition, corrected items, mean (s.d.)	105.7 (4.5)	108.2 (3.4)	108.8 (3.3)	105.4 (5.6)	106.9 (4.6)	107.5 (3.6)	103.3 (8.2)	105.5 (6.1)	108.7 (2.8)
*Clinical symptoms*									
Sample size	45	38	23	40	31	22	39	33	18
PANSS total, mean (s.d.)	47.5 (15.4)	37.6 (9.3)	39.1 (9.7)	46.4 (15.9)	39.1 (10.9)	35.6 (6.3)	45.9 (15.3)	49.4 (15.8)	42.2 (10.7)
PANSS positive, mean (s.d.)	10.2 (3.7)	8.7 (2.7)	8.6 (2.2)	9.5 (4.5)	8.3 (2.4)	7.8 (1.5)	9.9 (3.8)	10.1 (3.5)	9.9 (4.4)
PANSS negative, mean (s.d.)	10.8 (4.2)	8.5 (2.7)	8.4 (3.1)	10.9 (5.6)	9.5 (3.7)	8.1 (2.1)	11.2 (4.1)	12.7 (5.1)	9.6 (3.8)
PANSS general, mean (s.d.)	26.6 (9.3)	20.4 (5.5)	22.1 (7.0)	26.0 (9.2)	21.3 (6.7)	19.6 (3.9)	24.8 (9.2)	26.6 (9.6)	22.7 (5.2)
CDS total, mean (s.d.)	4.1 (4.2)	1.7 (2.4)	2.7 (3.4)	3.3 (4.0)	1.6 (2.3)	1.7 (1.9)	3.7 (4.7)	4.3 (4.3)	1.8 (1.7)
*Hippocampal volume (mm* ^*3*^)									
Sample size	25	20		28	17		20	13	
Left HPC, mean (s.d.)	3,511 (290)	3,459 (259)		3,461 (385)	3,475 (428)		3,533 (314)	3,530 (272)	
Right HPC, mean (s.d.)	3,726 (309)	3,667 (262)		3,645 (373)	3,638 (378)		3,726 (326)	3,724 (310)	
Total HPC, mean (s.d.)	7,237 (589)	7,126 (506)		7,106 (741)	7,113 (793)		7,259 (629)	7,254 (560)	
*Quality of Life*									
Sample size	44	38		40	31		38	32	
Physical health, mean (s.d.)	64.1 (16.9)	71.4 (18.0)		64.5 (17.2)	75.0 (17.4)		69.4 (17.4)	68.8 (19.5)	
Psychological health, mean (s.d.)	57.2 (22.0)	67.8 (21.6)		60.0 (19.2)	73.1 (29.6)		60.7 (20.0)	57.2 (23.6)	
*Body perception*									
Sample size	44	38	23	40	31	22	38	32	18
FRS, mean (s.d.)	1.8 (1.2)	1.8 (1.4)	2.0 (1.1)	1.5 (1.0)	1.5 (1.2)	1.6 (1.3)	1.6 (1.4)	1.3 (1.3)	1.5 (1.3)
*Drug adherence*									
Sample size	41	37	23	37	31	22	35	32	18
MCR, mean (s.d.)	6.0 (0.7)	6.0 (0.5)	5.9 (0.4)	5.9 (0.8)	6.1 (0.7)	6.0 (0.8)	5.9 (0.5)	6.0 (0.7)	5.9 (0.3)
*Fitness*									
Sample size	38	23		35	18		23	11	
VO_2_ max	25.3 (6.2)	25.9 (5.5)		27.1 (5.4)	29.2 (5.9)		26.1 (5.0)	26.0 (5.2)	

Abbreviations: CDS, calgary depression scale; FRS, figure rating scale; HKLLT, Hong Kong list learning test; HPC, hippocampus; MCR, medication compliance rating; PANSS, positive and negative syndrome scale.

**Table 2:** Statistical significance and associated effect sizes of comparisons within and between the groups over 12 weeks^a^

	*Yoga and Control Groups*					*Aerobic exercise and Control Groups*				
	*Time*		*Group×Time*		*ES (Cohen’d)*	*Time*		*Group×Time*		*ES (Cohen’d)*
	*F*	*P value*	*F*	*P value*		*F*	*P value*	*F*	*P value*	
Verbal acquisition	37.24	<0.010	10.81	<0.010	0.97	21.19	<0.010	5.72	0.020	0.83
Verbal retention	37.81	<0.010	5.22	.026	0.40	29.41	<0.010	6.20	0.016	0.56
DS forwards test	5.71	0.020	9.56	<0.010	0.77	2.65	0.109	6.43	0.014	0.59
DS backwards test	2.79	0.100	10.75	<0.010	0.71	13.60	<0.010	30.82	<0.010	1.08
LC Q score	3.86	0.054	7.45	<0.010	0.69	1.08	0.303	3.13	0.082	0.22
Stroop test congruent condition, correct items	1.81	0.183	2.00	0.162	0.34	0.90	0.347	0.89	0.349	0.30
Stroop test incongruent condition, correct items	8.50	<0.010	0.28	0.597	0.12	4.61	0.035	1.13	0.292	0.36
PANSS total	1.02	0.316	11.87	<0.010	1.54	0.11	0.747	6.76	0.012	1.14
PANSS positive	0.87	0.354	2.80	0.099	0.51	0.88	0.351	3.37	0.071	0.29
PANSS negative	0.04	0.838	11.70	<0.010	0.91	0.75	0.392	3.50	0.067	0.61
PANSS general	1.36	0.249	11.09	<0.010	0.95	0.35	0.556	6.63	0.013	0.32
CDS total	0.58	0.451	10.38	<0.010	0.74	0.38	0.538	8.79	<0.010	0.58
QoL (physical health)	0.91	0.343	6.21	0.015	0.46	4.35	0.042	15.52	<0.010	0.62
QoL (psychological health)	0.95	0.333	9.37	<0.010	0.65	1.29	0.261	6.36	0.015	0.73
Body perception (FRS)	1.69	0.198	1.14	0.288	0.37	2.43	0.124	0.32	0.577	0.23
Drug adherence (CRS)	0.08	0.786	0.41	0.525	0.21	1.30	0.259	0.06	0.816	0.13
VO_2_ max	0.25	0.624	0.12	0.729	0.24	4.08	0.054	0.35	0.560	0.22

Abbreviations: CDS, calgary depression scale; CRS, compliance rating scale; DS, digit span test; ES, effect size; FRS, figure rating scale; LC, letter cancellation test; PANSS, positive and negative syndrome scale; QoL, quality of life.

a *P* values represent the statistical significance of the effect of time and of the Group×Time interaction in a mixed model in unstructured variance matrix. Age, education years, length of illness, and antipsychotic dose were included as covariates for cognitive data analysis, and age, length of illness and antipsychotic dose were included as covariates for clinical data analysis.

**Table 3:** Assessments used at baseline and 12 weeks

	*Measures*	
Physical fitness	VO_2_ max test	Maximum capacity of an individual’s body to transport and utilize oxygen during incremental exercise.
Cognitive function	Hong Kong list learning test (HKLLT)	Verbal memory
	Digit span test	Working memory
	Letter cancellation test	Attention
	Stroop color and word tests	Executive function
Clinical symptoms	Positive and negative syndrome scale (PANSS)	Clinical symptoms
	Calgary depression scale (CDS)	Depressive symptoms for schizophrenia
Brain structures	Structural magnetic resonance imaging (MRI)	—
Body perception	Figure rating scale (FRS)	—
Medication adherence	Compliance rating scale (CRS)	—
Quality of life	The short form (36) health survey (SF-36)	—

**Table 4:** Demographic information for subjects participating in the study

	*Mean (s.d.)*		
	*Yoga group (n*=*45)*	*Aerobic exercise group (n*=*40)*	*Waitlist control group (n*=*39)*
*Demographic characteristics*			
* *Age, y	23.8 (6.8)	24.6 (7.9)	25.3 (8.1)
* *Education, y	12.0 (2.1)	13.1 (3.3)	12.2 (2.6)
* *Length of illness, y	2.5 (2.1)	2.4 (2.0)	2.0 (2.0)
* *Smoking, no. (%)			
* *Never smoker	42 (93.3)	35 (87.5)	38 (97.4)
* *Past smoker	0	1 (2.5)	0
* *Current smoker	3 (6.7)	4 (10.0)	1 (2.6)
* *Substance abuse, no. (%)			
* *No substance abuse	45 (100)	40 (100)	39 (100)
* *Substance abuse	0	0	0
* *Diagnosis, no. (%)			
* *Schizophrenia	21 (47.7)	20 (50.0)	19 (50.0)
* *Schizoaffective disorder	4 (9.1)	6 (15.0)	3 (7.9)
* *Schizophreniform disorder	0	1 (2.5)	0
* *Brief psychosis	2 (4.5)	3 (7.5)	0
* *Psychosis NOS	16 (36.4)	8 (20.0)	15 (39.5)
* *Delusional disorder	1 (2.2)	2 (5.0)	1 (2.6)
* *Antipsychotic medication dose, CPZ	339 (263)	288 (240)	260 (211)
* *Antipsychotic medication, no. (%)			
*First generation antipsychotics*			
* *Haloperidol	0	0	1 (2.9)
* *Flupenthixol	0	0	1 (2.9)
* *Zuclopenthixol	2 (4.4)	1 (2.6)	0
* *Perphenazine	0	0	1 (2.9)
* *Trifluoperazine	2 (4.4)	0	0
*Second generation antipsychotics*			
* *Clozapine	3 (7.0)	3 (7.7)	0
* *Risperidone	8 (18.6)	11 (28.2)	12 (34.3)
* *Olanzapine	11 (25.6)	7 (17.9)	6 (17.1)
* *Quetiapine	9 (20.9)	4 (10.3)	7 (20.0)
* *Amisulpiride	4 (8.9)	3 (7.7)	2 (5.7)
* *Ziprasidone	0	1 (2.6)	0
* *Aripiprazole	3 (7.0)	8 (20.5)	5 (14.3)
* *Sulpiride	0	1 (2.6)	0

*Clinical symptoms*			
* PANSS score*			
* *Total	47.51 (15.4)	46.35 (15.9)	45.87 (15.3)
* *Positive	10.2 (3.7)	9.5 (4.5)	9.9 (3.8)
* *Negative	10.8 (4.2)	10.9 (5.6)	11.2 (4.1)
* *General	26.6 (9.3)	26.0 (9.2)	24.8 (9.2)
CDS total score	4.1 (4.2)	3.3 (4.0)	3.7 (4.7)

Abbreviations: CPZ, chlorpromazine; CDS, calgary depression scale; PANSS, positive and negative syndrome scale; y, years.
