# Supplementary material for: Aerobic exercise and yoga improve neurocognitive function in women with early psychosis
Source: NPJ Schizophr. 2015 Dec 2;1:15047. doi: 10.1038/npjschz.2015.47 (PMC4849465; doi:10.1038/npjschz.2015.47)
Supplement: Supplementary Information [file npjschz201547-s1.doc]

**Supplementary Information**

Measures

*Fitness Testing:* Patients started walking on the treadmill (Full Vision Inc., model TMX425C TRACKMASTER®) with the fixed speed of 5.3 km/h with incline increments of 1 degree every minute until volitional exhaustion was reached. Subjects wore a mask to capture and record the inspired and expired air in order to determine the consumption of oxygen at different effort levels with a Medgraphics Ultima™ CardiO2 system (Medical Graphics Corporation, St Paul, MN). During the test procedure heart rate was monitored throughout, while measurements of VO2, carbon dioxide output (VCO2), and minute ventilation took place every 60 seconds. VO_2_ max is the maximum capacity of an individual's body to transport and utilize oxygen during incremental exercise, which reflects physical fitness. VO_2_ max is reached when oxygen consumption remains at steady state despite an increase in workload.

*Cognitive Testing:* The primary outcomes included verbal memory, working memory and attention. Verbal memory was measured using the validated Hong Kong List Learning Test (HKLLT)^1^, suitable for Cantonese-speakers. The test included random and blocked conditions. In each condition, a list of 16 two-character Chinese phrase was read to the participant three times. The words in the “random” condition were arranged randomly with the condition; no two items from the same category were presented consecutively. The words in the “blocked” condition were organized into clusters based upon categories. The participant was asked to recall the same list after 10 (short-term delay) and 30 minutes (long-term delay). The number of words correctly recalled was recorded and scores of three trials were summed as an index of “verbal acquisition”. The scores of short-term and long-term delayed recall were summed as an index of “verbal retention”. Recognition test consisted of the 16 target words interspersed among 16 distracter items. Working memory was tested by Digit Span test, a subtest of the Wechsler Adult Intelligence Scale-Revised. It includes digit-span forwards (DF) and digit-span backwards (DB) tests. The participant was asked to recall a series of numbers in the same order in the DF test, and in reverse order in the DB test. Each contains 14 sequences of numbers. The correctly recalled series were scored as 1. The Letter Cancellation Test was used to evaluate attention and concentration.^2^ The basic version of the task consists of six 52-character rows in which the target character is randomly interspersed approximately 18 times in each row. Subjects were asked to cancel the letter “C” and “E” as quickly as possible. The time to completion, number of error and omission items were recorded.^2^ A “quality of search” index (Q), developed by Geldmacher et al., was applied for the analysis. Q is the ratio of correct number to total number of targets multiplied by the ratio of correct number per time unit. Higher Q scores represent more efficient performance and better attention and concentration.^3^ Stroop Color and Word Tests in both congruent and incongruent conditions were used to test executive function. The number of corrected items and response time were recorded for analysis.^4^

*Clinical Assessments:* Symptom severity in patients was measured with the PANSS. Depressive symptoms were measured with the CDS, which was developed specifically to measure depression in schizophrenia.

*Structural Magnetic Resonance Imaging (MRI)*: Randomized participants in each group participated in imaging studies until at least 20 per group had completed baseline scans free of motion artifact for analysis. Participants were imaged at 3T (Philips Achieva 3T Quasar). A T1-weighted, MPRAGE sequence (TE=3.2ms, TR=7ms, flip angle=8°, FOV 240mm×240 mm) of 155 consecutive slices was acquired with a sagittal orientation and a voxel size of 1mm×1mm×1 mm. All T1-weighted structural MRI scans were bias-corrected and initially segmented into CSF, GM and WM using FSL FAST.^5^ Multi-atlas segmentation (MAS) with joint label fusion was then used to segment left and right hippocampus for each scan.^6^ Errors produced by label transfer were further reduced by joint label fusion.

*Figure Rating Scale (FRS):* is used to measure body perception. There are seven male/female contour drawings, numbered “1” to “7”, in increasing body size from left to right. Participants were asked to choose one figure drawing that most accurately represents the size of their own bodies and then one that represents their desired figure. Dissatisfaction with body figure was expressed as the difference between the two chosen ratings.

*Compliance Rating Scale (CRS):* is used to assess medication adherence. The scale consists of ratings from 1-7, 1 indicating complete refusal of medication while 7 indicates very good adherence in which the patient actively participates, readily accepts and shows some responsibility for the regimen.

*The Short Form (36) Health Survey (SF-36):* a survey of quality of life consisting of 8 scaled scores, which are the weighted sums of the questions in their section, covering physical and psychological aspects.

Intervention programs

*Integrated yoga therapy*: Classic Hatha yoga was selected for its wide application in previous studies and comprehensive effects on the body and mind. It was also an easy-learning basic yoga style, and was practical in patients with psychosis who had poorer physical function than healthy people. Hatha yoga is a classic yoga style including breathing practice, body postures and relaxation. The meditation component was excluded, as possible adverse effects on acute psychosis were reported in some case studies.^27^ Each session consisted of breathing control (10 minutes), body postures (40-45 minutes), and relaxation (5 minutes). Each yoga class of 5 to 10 participants was conducted by a qualified yoga instructor. The yoga training program aimed to create an overall harmony between body and mind. The postures were designed to cover all body parts to give the body overall strength and flexibility, with some modifications based on individuals physical conditions. Each posture was maintained for 3-5 breaths. The yoga classes were taught by a qualified yoga instructor with a PhD degree.

*Aerobic exercise program:* Included walking on a treadmill (15-20 minutes), and stationary cycling (25-30 minutes) followed by cool-down stretching (5 minutes). Each subject’s heart rate was continuously monitored during the exercise sessions using a portable recorder, to ensure that the participant maintained a moderate intensity exercise exertion level (50%-60% of the VO_2_ max value). Each aerobic exercise training group size consisted of not more than 10 participants, and was supervised by a qualified exercise physiologist with a Master degree.

REFERENCES

1 Chan, A. S., Kwok, I. *Hong Kong List Learning Test*. (Department of Psychology, CUHK, 1999).

2 Diller, L. *et al.* *Studies in cognition and rehabilitation in hemiplegia*. (1974).

3 Geldmacher, D. S. & Hills, E. C. Effect of stimulus number, target-to-distractor ratio, and motor speed on visual spatial search quality following traumatic brain injury. *Brain Injury* **11**, 59-66 (1997).

4 Stroop, J. R. Studies of interference in serial verbal reactions. *Journal of Experimental Psychology* **18**, 643-662 (1935).

5 Zhang, Y., Brady, M. & Smith, S. Segmentation of brain MR images through a hidden Markov random field model and the expectation-maximization algorithm. *IEEE transactions on medical imaging* **20**, 45-57, doi:10.1109/42.906424 (2001).

6 Wang, H. *et al.* Multi-Atlas Segmentation with Joint Label Fusion. *IEEE transactions on pattern analysis and machine intelligence*, doi:10.1109/TPAMI.2012.143 (2012).
